# Complete mitochondrial genome of a livebearing freshwater fish (Cyprinodontiformes: Poeciliidae): *Poecilia parae*

**DOI:** 10.1080/23802359.2023.2171246

**Published:** 2023-02-05

**Authors:** Kayla M. Fast, Alex W. Rakestraw, Michael W. Sandel

**Affiliations:** a Department of Biological and Environmental Sciences, The University of West Alabama, Livingston, AL, USA; b Department of Wildlife, Fisheries, and Aquaculture, Mississippi State University, Mississippi State, MS, USA

**Keywords:** *Poecilia parae*, Oxford Nanopore, mitochondrial genome, Poeciliidae

## Abstract

Members of the fish family Poeciliidae (livebearing ‘tooth-carps’) have historically been used as models in medical research, behavior ecology, and biological control. This group of primarily freshwater fishes is highly tolerant to environmental factors such as salinity and warm temperatures and includes some invasive species. Here, we present the mitochondrial genome of *Poecilia parae*. A representative of this species was obtained from Suriname. The complete mitochondrial genome was sequenced using Oxford Nanopore technology and is 16,559 bp long. The genome contains 13 protein-coding genes, two ribosomal RNAs (rRNAs), 22 transfer RNAs (tRNAs), and one control region (D-loop). Phylogenetic analysis yielded topologies similar to those previously published. The data generated here will be useful in future studies of comparative biology and those utilizing environmental DNA (eDNA).

## Introduction

For many decades, livebearing fishes of the family Poeciliidae have been valuable models for research in evolutionary ecology and comparative biology. Specifically, the Guppy (*Poecilia reticulata*) and Southern Platyfish (*Xiphophorus maculatus*) have served as indicator taxa and as models for behavioral ecology, life history evolution, and cancer biology (Schartl 2014; Reznick et al. 2017; Goldberg et al. 2019; Gomes-Silva et al. 2020). Poeciliids, including some invasive species, can use a wide range of habitats because they are successful colonizers and have high thermal and salinity tolerances (Meffe and Snelson 1989). Here, we present the mitochondrial genome of a lesser-known species with close phylogenetic affinity to *P. reticulata*, *P. parae*. We anticipate that the mitogenome presented here will aid future research in comparative biology and will be useful for noninvasive investigations of watersheds using environmental DNA (eDNA).

**Figure 1.**
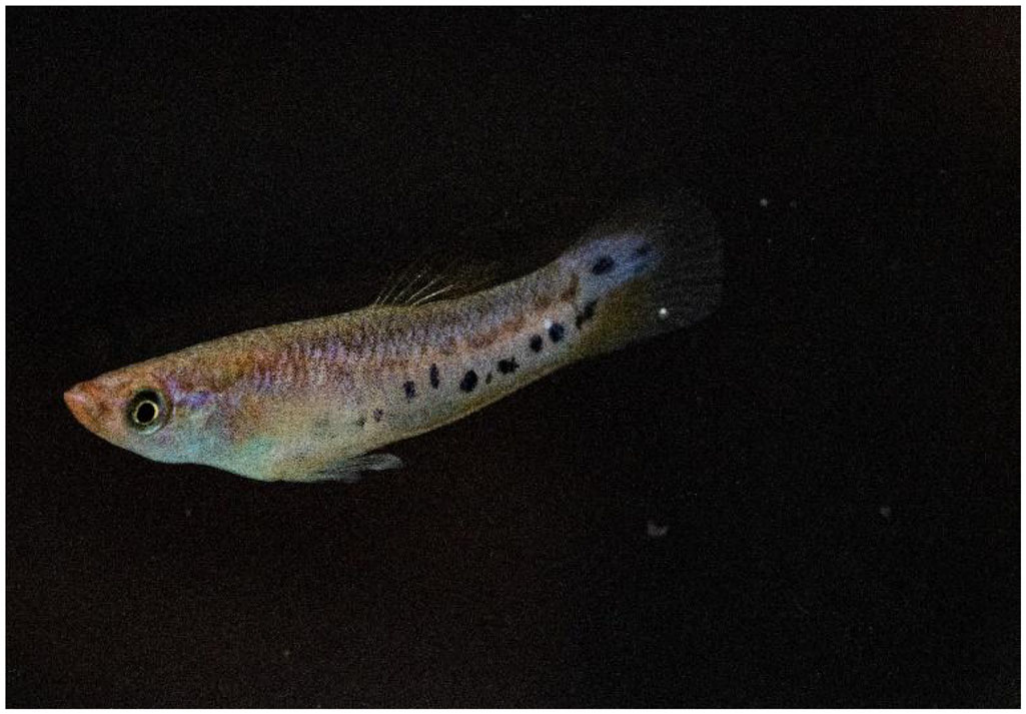
Representative photograph of *Poecilia parae* (blue melanzona morph).

*Poecilia parae* (Eigenmann, 1894) occupies a geographic range from Guyana to northern Brazil (Figure 1). *Poecilia parae* is a novel model system for the study of sex chromosome evolution and sexual polymorphism (Metzger et al. 2021; Sandkam et al. 2021). The International Union for Conservation of Nature (IUCN) has not evaluated the conservation status of *P. parae*. Congeners of *Poecilia* in the genus *Xiphophorus* are important models for the study of sexual dimorphism, sex chromosome evolution, and carcinogenesis (Schartl 1990; Woolcock et al. 2006; Schartl and Walter 2016). While many studies have examined the evolutionary history of Poeciliids in the contexts of ornamentation and sexual selection, few have used complete mitochondrial data (Morris et al. 2001; Cui et al. 2013; Kang et al. 2013; Goldberg et al. 2019; Méndez-Janovitz et al. 2019; Metzger et al. 2021; Sandkam et al. 2021).

## Materials and methods

An aquarium trade specimen of *Poecilia parae* (blue melanzona morph) was obtained from Suriname (5°51′36″N, 55°7′48″W). The preserved specimen was deposited in the University of West Alabama Zoological Collection (https://www.uwa.edu/, kaylafast0@gmail.com) under voucher number AR20090201:03. Whole genomic DNA was extracted from the pectoral fin using the DNeasy Blood and Tissue Kit following the manufacturer’s instructions (QIAGEN, Hilden, Germany). DNA quality was confirmed by gel electrophoresis using a 1.5% agarose gel stained with ethidium bromide. The quantity of DNA was determined using a NanoDrop 2000 Spectrophotometer (Thermo Fisher Scientific, Waltham, MA). Purified DNA was stored at 4 °C.

The sequencing library was prepared using the Oxford Nanopore Ligation Sequencing Kit and loaded onto a Flongle flow cell following the manufacturer’s instructions (Oxford Nanopore, Oxford, UK). Sequencing was performed on a MinION device using the Flongle adapter and monitored with MinKNOW software v.22.08.9 (Figure S1; Oxford Nanopore, Oxford, UK). Basecalling was done in Guppy v.6.2.11 using the high-accuracy basecalling model and reads filtered to a minimum qscore = 9. Reads were assembled using Geneious Prime v.2022.2.2 under the Medium/Fast sensitivity setting and iterative fine-tuning. The *P. reticulata* mitochondrial genome (KJ460033) was selected as a reference sequence. A consensus sequence was generated using a strict 50% threshold and then checked by eye and ambiguous base calls resolved in BioEdit v.7.2.5 (Hall 1999; Hall and Alzohairy 2011). The genome was annotated in MitoAnnotator v.3.75 (Iwasaki et al. 2013; Sato et al. 2018). The presence of appropriate start and stop codons in protein coding genes was confirmed and internal stop codons resolved in MEGA11: Molecular Evolutionary Genetics Analysis v.11.0.10 (Tamura et al. 2021). The annotated mitochondrial genome is openly available in GenBank of NCBI at https://www.ncbi.nlm.nih.gov (OP326603). Congeneric species were identified using NCBI BLAST (Altschul et al. 1990; Miya et al. 2003; Setiamarga et al. 2008; Bai et al. 2009; Dang et al. 2016; Jeon et al. 2016; Jiang et al. 2016; Kong et al. 2016; Künstner et al. 2016; Sung et al. 2016; Zhang et al. 2016; Mateos et al. 2019; van Kruistum et al. 2020; Eastis et al. 2021). Concatenated protein-coding sequences from the congener mitochondrial genomes and a *Xenotoca eiseni* outgroup were aligned using the MAFFT server v.7 before phylogenetic analysis (Katoh et al. 2002; Katoh and Standley 2013). Model selection and evolutionary analysis by the maximum-likelihood method were performed in MEGA11. A maximum-likelihood phylogenetic tree was reconstructed using the general time reversible model with gamma and invariable sites allowed and 1000 bootstrap replications.

## Results

**Figure 2.**
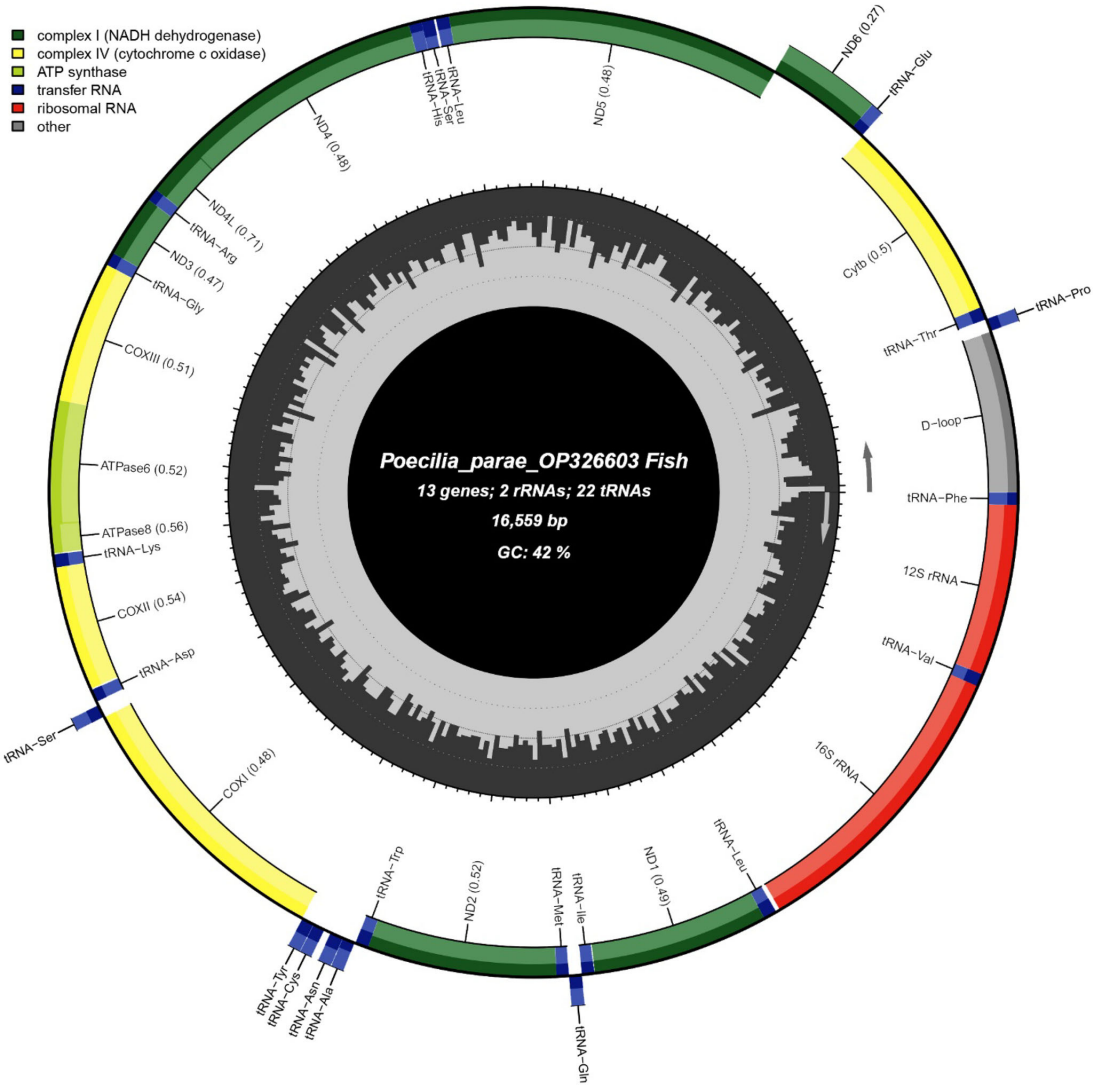
Mitochondrial genome map of *Poecilia parae.* The innermost circle of the image represents %GC per every 5 bp of the mitogenome; longer lines indicate higher %GC.

The mitochondrial genome of *P. parae* is 16,559 bp long. The nucleotide composition of the *P. parae* mitochondrial genome is 29.70% A, 27.25% C, 14.80% G, and 28.26% T. The genome is circular, consisting of 13 protein-coding genes, two ribosomal RNAs (rRNAs), 22 transfer RNAs (tRNAs), and one control region (D-loop; Figure 2). The *P. parae* mitochondrial genome contains 29 forward genes and nine reverse genes; all protein-coding genes use the start codon ATG. Seven protein-coding genes in the *P. parae* mitochondrial genome (ND1, COI, ATP8, ND4L, ND5, ND6, and CYTB) end with the complete TAA stop codon and six (ND2, COII, ATP6, COIII, ND3, and ND4) end with an incomplete stop codon which is completed by the addition of 3′ A residues.

## Discussion

**Figure 3.**
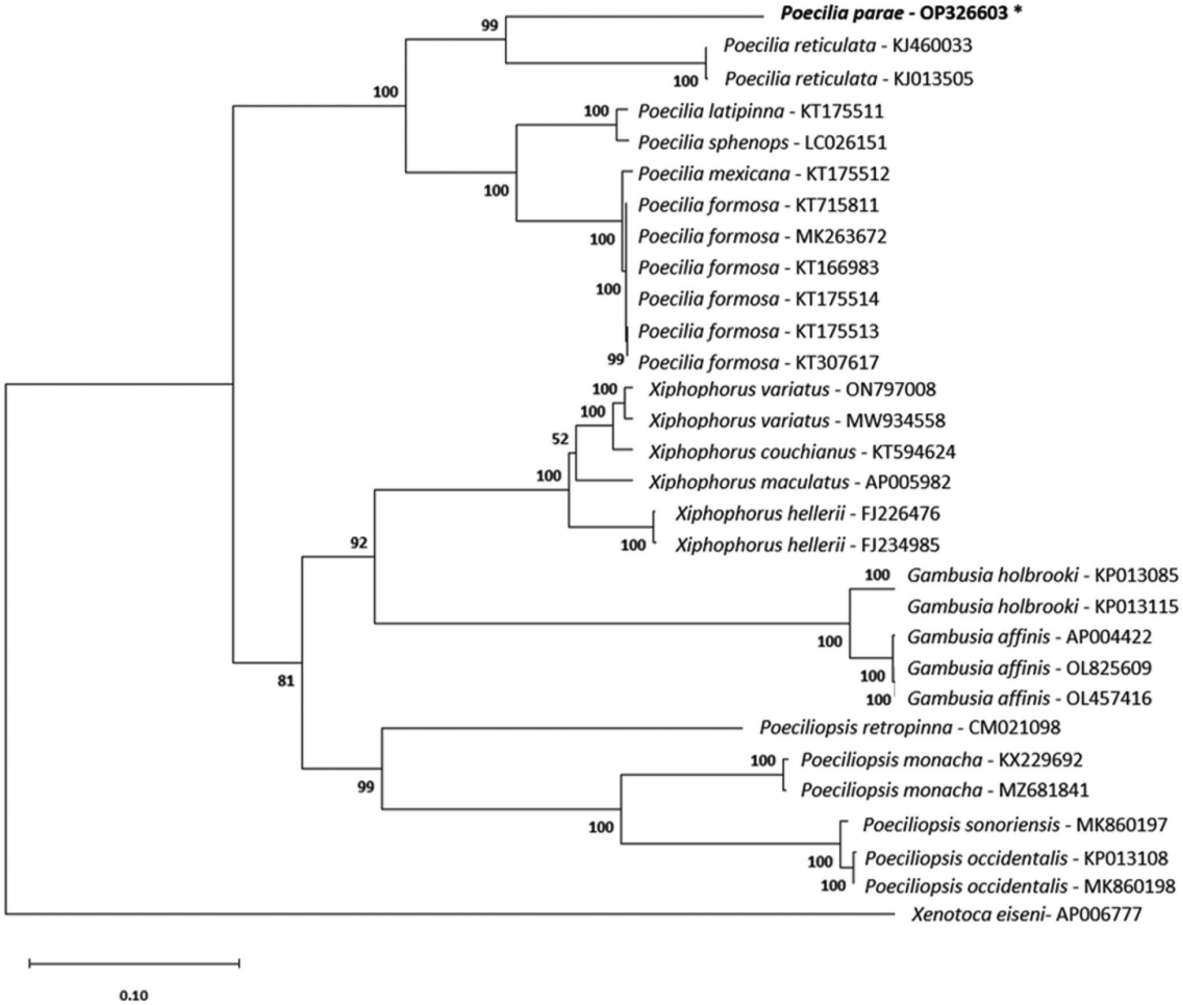
Maximum-likelihood phylogeny reconstructed using mitochondrial protein-coding sequences under the GTR + G+I model and 1000 bootstrap replicates. The following sequences were used: AP005982 (Miya et al. 2003), KT594624 (Zhang et al. 2016), MW934558 (Eastis et al. 2021), ON797008, FJ226476 (Bai et al. 2009), FJ234985 (Bai et al. 2009), CM021098 (van Kruistum et al. 2020), KJ013505 (Kong et al. 2016), KJ460033 Künstner et al. (2016), OP326603 (this study), KT166983 (Dang et al. 2016), KT175513, KT307617 (Sung et al. 2016), KT715811, MK263672, KT175514, KT175512, KT175511, LC026151 (Jiang et al. 2016), KX229692 (Jeon et al. 2016), MZ681841, MK860197 (Mateos et al. 2019), KP013108, MK860198 (Mateos et al. 2019), OL825609, OL457416, AP004422 (Miya et al. 2003), KP013085, KP013115, and AP006777 (Setiamarga et al. 2008). Numbers on nodes are bootstrap support values. The sequence generated in this study is written in bold font and marked with an asterisk.

Phylogenetic analysis using the maximum-likelihood method places the genera *Xiphophorus*, *Poecilia*, *Gambusia*, and *Poeciliopsis* each as monophyletic groups (Figure 3). Our data place *P. parae* as the sister group to *P. reticulata*, the Guppy. The phylogenetic tree topology of poeciliid genera is consistent with recent phylogenetic studies performed on whole poeciliid mitochondrial genomes (Pollux et al. 2014; Jeon et al. 2016; Eastis et al. 2021) and one-to-one orthologs (Mateos et al. 2019). Previous phylogenetic studies conducted with a more exhaustive sampling of *Poecilia* support the placement of *P. parae* (Pollux et al. 2014; Méndez-Janovitz et al. 2019; Metzger et al. 2021; Sandkam et al. 2021). A wider representation of *Poecilia* spp. in complete mitochondrial data will further resolve the positions of these taxa. The mitochondrial genome that we generated will be conducive to monitoring species presence using eDNA and aid in future research in comparative biology.

## Supplementary Material

Supplemental Material

## Data Availability

The data that support the findings of this study are openly available in GenBank of NCBI at https://www.ncbi.nlm.nih.gov, reference number OP326603. The associated BioProject, SRA, and BioSample numbers are PRJNA742674, SRR21296950, and SAMN30561436, respectively.

## References

[CIT0001] Altschul SF, Gish W, Miller W, Lipman DJ. 1990. Basic local alignment search tool. J Mol Biol. 215:403–410. 2231712 10.1016/S0022-2836(05)80360-2

[CIT0002] Bai J-J, Liu Y-F, Quan Y-C, He X-P, Ye X. 2009. The complete mitochondrial genome of the RR-B strain of swordtail (*Xiphophorus hellerii*). Mitochondrial DNA. 20(4):72–77. 19575330 10.1080/19401730903033105

[CIT0003] Cui R, Schumer M, Kruesi K, Walter R, Andolfatto P, Rosenthal GG. 2013. Phylogenomics reveals extensive reticulate evolution in *Xiphophorus* fishes. Evolution. 67(8):2166–2179. 23888843 10.1111/evo.12099

[CIT0004] Dang X, Xia Y, Xu Q, Zhang J. 2016. The complete mitochondrial genome of the *Poecilia formosa* (Amazon molly). Mitochondrial DNA A DNA Mapp Seq Anal. 27:3523–3524. 26260185 10.3109/19401736.2015.1074200

[CIT0005] Eastis AN, Fast KM, Sandel MW. 2021. The complete mitochondrial genome of the Variable Platyfish *Xiphophorus variatus*. Mitochondrial DNA B Resour. 6(9):2640–2642. 34409164 10.1080/23802359.2021.1963339 PMC8366644

[CIT0006] Goldberg DL, Landy JA, Travis J, Springer MS, Reznick DN. 2019. In love and war: the morphometric and phylogenetic basis of ornamentation, and the evolution of male display behavior, in the livebearer genus *Poecilia*. Evolution. 73(2):360–377. 30652306 10.1111/evo.13671

[CIT0007] Gomes-Silva G, Pereira BB, Liu K, Chen B, Santos VSV, de Menezes GHT, Pires LP, Santos BMT, Oliveira DM, Machado PHA, et al. 2020. Using native and invasive livebearing fishes (Poeciliidae, Teleostei) for the integrated biological assessment of pollution in urban streams. Sci Total Environ. 698:134336. 31783440 10.1016/j.scitotenv.2019.134336

[CIT0008] Hall T. 1999. BioEdit: a user-friendly biological sequence alignment editor and analysis program for Windows 95/98/NT. Nucleic Acids Symp Series. 41:95–98.

[CIT0009] Hall T, Alzohairy DAM. 2011. BioEdit: an important software for molecular biology. GERF Bull Biosci. 2:60–61.

[CIT0010] Iwasaki W, Fukunaga T, Isagozawa R, Yamada K, Maeda Y, Satoh TP, Sado T, Mabuchi K, Takeshima H, Miya M, et al. 2013. MitoFish and MitoAnnotator: a mitochondrial genome database of fish with an accurate and automatic annotation pipeline. Mol Biol Evol. 30(11):2531–2540. 23955518 10.1093/molbev/mst141 PMC3808866

[CIT0011] Jeon YS, Johnson SB, Won Y-J, Vrijenhoek RC. 2016. Complete mitochondrial genome of the headwater livebearer, *Poeciliopsis monacha*: the mother of clones. Mitochondrial DNA B Resour. 1(1):793–794. 33473629 10.1080/23802359.2016.1197066 PMC7800649

[CIT0012] Jiang L, Zhang S, Chen B, et al. 2016. Complete mitochondrial genomes of two ornamental fishes. Mitochondrial DNA A DNA Mapp Seq Anal. 27:2531–2532. 26061340 10.3109/19401736.2015.1038790

[CIT0013] Kang JH, Schartl M, Walter RB, Meyer A. 2013. Comprehensive phylogenetic analysis of all species of swordtails and platies (Pisces: Genus *Xiphophorus*) uncovers a hybrid origin of a swordtail fish, *Xiphophorus monticolus*, and demonstrates that the sexually selected sword originated in the ancestral lineage of the genus, but was lost again secondarily. BMC Evol Biol. 13:25. 23360326 10.1186/1471-2148-13-25 PMC3585855

[CIT0014] Katoh K, Misawa K, Kuma K, Miyata T. 2002. MAFFT: a novel method for rapid multiple sequence alignment based on fast Fourier transform. Nucleic Acids Res. 30(14):3059–3066. 12136088 10.1093/nar/gkf436 PMC135756

[CIT0015] Katoh K, Standley DM. 2013. MAFFT multiple sequence alignment software version 7: improvements in performance and usability. Mol Biol Evol. 30(4):772–780. 23329690 10.1093/molbev/mst010 PMC3603318

[CIT0016] Kong X-F, Li J-T, Sun X-W. 2016. Complete mitochondrial genome of the guppy (*Poecilia reticulata*). Mitochondrial DNA A DNA Mapp Seq Anal. 27:228–229. 24495134 10.3109/19401736.2014.880902

[CIT0017] Künstner A, Hoffmann M, Fraser BA, Kottler VA, Sharma E, Weigel D, Dreyer C. 2016. The genome of the Trinidadian Guppy, *Poecilia reticulata*, and variation in the Guanapo population. PLOS One. 11(12):e0169087. 28033408 10.1371/journal.pone.0169087 PMC5199103

[CIT0018] Mateos M, Kang D, Klopp C, Parrinello H, García-Olazábal M, Schumer M, Jue NK, Guiguen Y, Schartl M. 2019. Draft genome assembly and annotation of the Gila Topminnow *Poeciliopsis occidentalis*. Front Ecol Evol. 7:404.

[CIT0019] Meffe GK, Snelson F. 1989. An ecological overview of poeciliid fishes. In: Meffe GK, Snelson F, editors. Ecology and evolution of livebearing fishes (Poeciliidae). New York: Prentice Hall; p. 13–31.

[CIT0020] Méndez-Janovitz M, Gonzalez-Voyer A, Macías Garcia C. 2019. Sexually selected sexual selection: can evolutionary retribution explain female ornamental colour? J Evol Biol. 32(8):833–843. 31070826 10.1111/jeb.13485

[CIT0021] Metzger D, Sandkam BA, Darolti I, Mank JE. 2021. Rapid evolution of complete dosage compensation in *Poecilia*. Genome Biol Evol. 13:1–7. 10.1093/gbe/evab155 PMC8325565 34240180

[CIT0022] Miya M, Takeshima H, Endo H, Ishiguro NB, Inoue JG, Mukai T, Satoh TP, Yamaguchi M, Kawaguchi A, Mabuchi K, et al. 2003. Major patterns of higher teleostean phylogenies: a new perspective based on 100 complete mitochondrial DNA sequences. Mol Phylogenet Evol. 26(1):121–138. 12470944 10.1016/s1055-7903(02)00332-9

[CIT0023] Morris MR, Queiroz KD, Morizot DC. 2001. Phylogenetic relationships among populations of Northern Swordtails (*Xiphophorus*) as inferred from allozyme data. Copeia. 2001:65–81.

[CIT0024] Pollux BJA, Meredith RW, Springer MS, Garland T, Reznick DN. 2014. The evolution of the placenta drives a shift in sexual selection in livebearing fish. Nature. 513(7517):233–236. 25043015 10.1038/nature13451

[CIT0025] Reznick DN, Furness AI, Meredith RW, Springer MS. 2017. The origin and biogeographic diversification of fishes in the family Poeciliidae. PLOS One. 12(3):e0172546. 28278162 10.1371/journal.pone.0172546 PMC5344339

[CIT0026] Sandkam BA, Almeida P, Darolti I, Furman BLS, van der Bijl W, Morris J, Bourne GR, Breden F, Mank JE. 2021. Extreme Y chromosome polymorphism corresponds to five male reproductive morphs of a freshwater fish. Nat Ecol Evol. 5(7):939–948. 33958755 10.1038/s41559-021-01452-w

[CIT0027] Sato Y, Miya M, Fukunaga T, Sado T, Iwasaki W. 2018. MitoFish and MiFish pipeline: a mitochondrial genome database of fish with an analysis pipeline for environmental DNA metabarcoding. Mol Biol Evol. 35(6):1553–1555. 29668970 10.1093/molbev/msy074 PMC5967551

[CIT0028] Schartl M. 1990. Homology of melanoma-inducing loci in the genus *Xiphophorus*. Genetics. 126(4):1083–1091. 1981761 10.1093/genetics/126.4.1083 PMC1204271

[CIT0029] Schartl M. 2014. Beyond the zebrafish: diverse fish species for modeling human disease. Dis Model Mech. 7(2):181–192. 24271780 10.1242/dmm.012245 PMC3917239

[CIT0030] Schartl M, Walter RB. 2016. *Xiphophorus* and medaka cancer models. Adv Exp Med Biol. 916:531–552. 27165369 10.1007/978-3-319-30654-4_23

[CIT0031] Setiamarga DHE, Miya M, Yamanoue Y, Mabuchi K, Satoh TP, Inoue JG, Nishida M. 2008. Interrelationships of Atherinomorpha (medakas, flyingfishes, killifishes, silversides, and their relatives): the first evidence based on whole mitogenome sequences. Mol Phylogenet Evol. 49(2):598–605. 18771739 10.1016/j.ympev.2008.08.008

[CIT0032] Sung C-H, Wu C-C, Tseng C-T, Lu J-K, Lin H-C. 2016. The complete mitochondrial genome of *Poecilia formosa* (Actinopterygii: Cyprinodontiformes: Poeciliidae). Mitochondrial DNA A DNA Mapp Seq Anal. 27:4331–4332. 26465834 10.3109/19401736.2015.1089487

[CIT0033] Tamura K, Stecher G, Kumar S. 2021. MEGA11: molecular evolutionary genetics analysis version 11. Mol Biol Evol. 38(7):3022–3027. 33892491 10.1093/molbev/msab120 PMC8233496

[CIT0034] van Kruistum H, Guernsey MW, Baker JC, Kloet SL, Groenen MAM, Pollux BJA, Megens H-J. 2020. The genomes of the livebearing fish species *Poeciliopsis retropinna* and *Poeciliopsis turrubarensis* reflect their different reproductive strategies. Mol Biol Evol. 37(5):1376–1386. 31960923 10.1093/molbev/msaa011 PMC7182214

[CIT0035] Woolcock B, Kazianis S, Lucito R, Walter RB, Kallman KD, Morizot DC, Vielkind JR. 2006. Allele-specific marker generation and linkage mapping on the *Xiphophorus* sex chromosomes. Zebrafish. 3(1):23–37. 18248244 10.1089/zeb.2006.3.23

[CIT0036] Zhang K, Liu S, Chen D-Y, Chen X, Zhang G-Y. 2016. The complete mitochondrial genome of the Monterrey platyfish (*Xiphophorus couchianus*). Mitochondrial DNA A DNA Mapp Seq Anal. 27:4453–4454. 26403180 10.3109/19401736.2015.1089567

