# Supplementary material for: Complete mitochondrial genome of a livebearing freshwater fish (Cyprinodontiformes: Poeciliidae): Poecilia parae
Source: Mitochondrial DNA B Resour. 2023 Feb 5;8(2):215–9. doi: 10.1080/23802359.2023.2171246 (PMC9904314; doi:10.1080/23802359.2023.2171246)
Supplement: Supplemental Material [file TMDN_A_2171246_SM1428.docx]

Supplementary Material


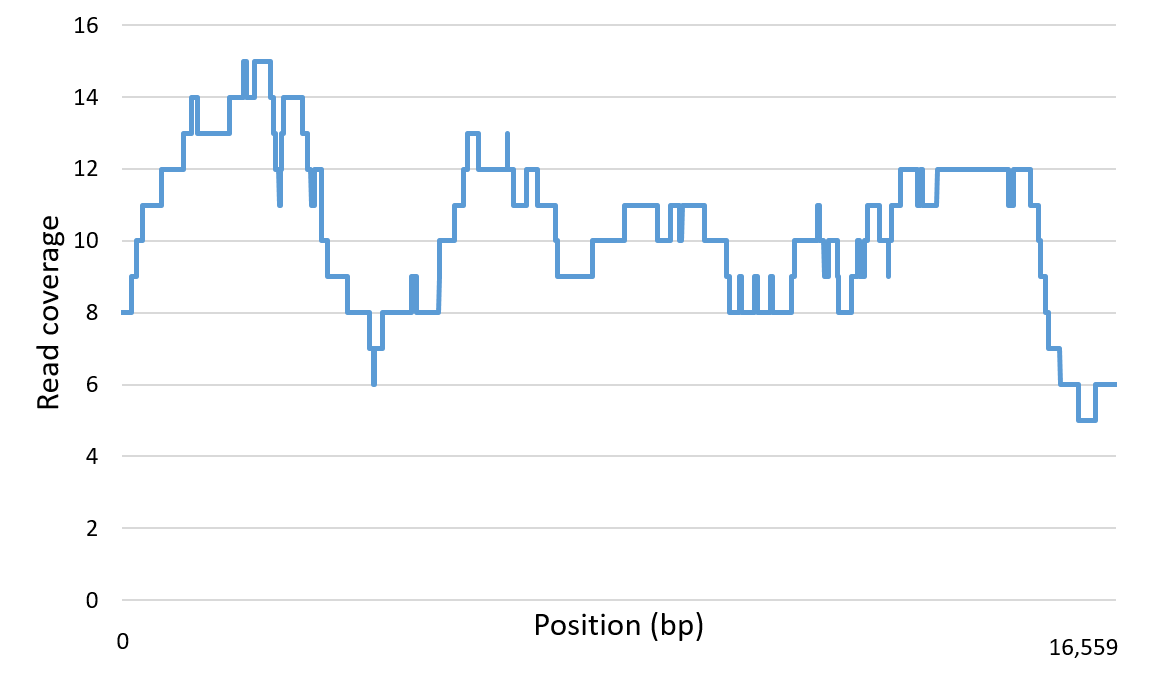


Figure S1: Depth of read coverage from mitochondrial genome sequencing.
